# Artificial covalent linkage of bacterial acyl carrier proteins for fatty acid production

**DOI:** 10.1038/s41598-019-52344-w

**Published:** 2019-11-05

**Authors:** Carlos Rullán-Lind, Melissa Ortiz-Rosario, Andrea García-González, Vivian Stojanoff, Nataliya E. Chorna, Ruth B. Pietri, Abel Baerga-Ortiz

**Affiliations:** 1 Department of Biochemistry, University of Puerto Rico, Medical Sciences Campus, San Juan, Puerto Rico 00936-5067 USA; 2 Molecular Sciences Research Center, University of Puerto Rico, San Juan, Puerto Rico USA; 3 NSLS II, Brookhaven National Laboratory, Upton, New York USA; 4 Department of Chemistry, University of Puerto Rico, Cayey Campus, Cayey, Puerto Rico USA

**Keywords:** Biocatalysis, Fatty acids, Metabolic engineering, Metabolic engineering

## Abstract

Acyl carrier proteins (ACPs) are essential to the production of fatty acids. In some species of marine bacteria, ACPs are arranged into tandem repeats joined by peptide linkers, an arrangement that results in high fatty acid yields. By contrast, *Escherichia coli*, a relatively low producer of fatty acids, uses a single-domain ACP. In this work, we have engineered the native *E*. *coli* ACP into tandem di- and tri-domain constructs joined by a naturally occurring peptide linker from the PUFA synthase of *Photobacterium profundum*. The size of these tandem fused ACPs was determined by size exclusion chromatography to be higher (21 kDa, 36 kDa and 141 kDa) than expected based on the amino acid sequence (12 kDa, 24 kDa and 37 kDa, respectively) suggesting the formation of a flexible extended conformation. Structural studies using small-angle X-ray scattering (SAXS), confirmed this conformational flexibility. The thermal stability for the di- and tri-domain constructs was similar to that of the unfused ACP, indicating a lack of interaction between domains. Lastly, *E*. *coli* cultures harboring tandem ACPs produced up to 1.6 times more fatty acids than wild-type ACP, demonstrating the viability of ACP fusion as a method to enhance fatty acid yield in bacteria.

## Introduction

Acyl carrier proteins (ACPs) are central to the production of fatty acids in all organisms^1^. They play a key role in the shuttling of substrates and intermediates during the successive rounds of malonate condensation and the subsequent modifications by ketoreductase, dehydratase and enoyl reductase enzymes that result in a full-length fatty acid^2^. At all times during the biosynthesis of a fatty acid, each starting material and intermediate is covalently linked to an ACP which carries the substrate from one active site to the next. ACPs are also important and serve homologous functions in other metabolic pathways such as the biosynthesis of polyketides by polyketide synthases (PKSs) and the biosynthesis of non-ribosomal peptides by non-ribosomal peptide synthases (NRPSs), all enzymes that employ a molecular logic similar to that of fatty acid biosynthesis^3^.

**Figure 1:**
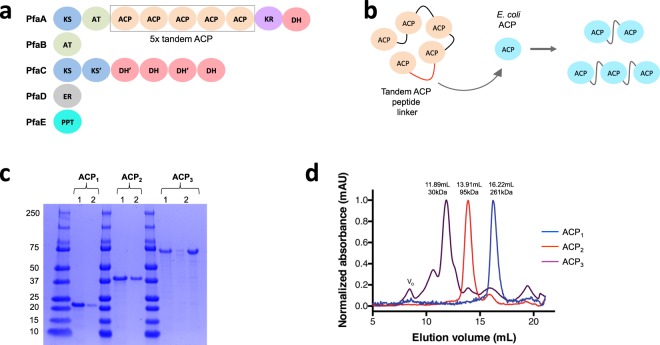
Construction of di and tri-domain tandem ACPs. To make polyunsaturated fatty acids some species of bacteria employ modular multi-enzymes known as PUFA synthases. (**a**) Although the PUFA synthase from *P*. *profundum* is shown here, the tandem arrangement of ACPs is a conserved feature of PUFA synthases. (**b**) To mimic the tandem architecture observed of natural systems we linked the native *E*. *coli* ACP using the natural peptide linker from the *P*. *profundum* PUFA synthase. (**c**) The ACP_1_, ACP_2_ and ACP_3_ were produced in *E*. *coli*, purified to homogeneity and analyzed by SDS -PAGE (each against a set of size markers). (**d**) The size of each construct was ascertained by size exclusion chromatography.

While ACPs typically function as independent proteins in most prokaryotes, there are some species that contain multiple covalently linked ACP domains in tandem^4,5^. The presence of tandem ACP domains has been reported in synthase systems involved in the production of several important natural products, for example, the polyunsaturated fatty acids (PUFA) as well as some polyketides^6–8^. Many PUFA synthases contain between 2 and 9 tandem ACP domains^8–10^. For instance, the PUFA synthase of *Photobacterium profundum* contains 5 covalently linked ACP domains^4,5^ (Fig. 1a). It has also been shown that the PKS that makes mupirocin in *Pseudomonas fluorescens* also contains tandem duplications of ACP, although most known PKSs contain a single ACP in each elongation module^11–13^.

The advantages of this tandem ACP arrangement in synthase systems have been reported for both the biosynthesis of fatty acids and polyketide antibiotics. In PUFA synthases, the yields of PUFA increase proportionally with the number of functional ACPs. A previous study showed that ACP domains are functionally equivalent regardless of their physical location and that production enhancement results from the proximity of ACPs in the arrangement^14^. Other studies have confirmed this result by showing an increase in PUFA yields with the insertion of either inactivated or active ACP domains from the pfaA multidomain proteins. The pfaA subunits which harbor these ACP domains and their linkers have been shown to be exchangeable between organisms^15^. Previous work by our group elucidated the solution structure of tandem ACPs from *P*. *profundum*, demonstrating that they have an elongated beads-on-a-string arrangement with flexible linkers with little or no interaction between domains^9^.

Like most ACPs, the ACP from *Escherichia coli* is a stand-alone monomer, which has been demonstrated to be toxic to *E*. *coli* cells when overexpressed^16^. Thus, strategies to increase fatty acid production by overexpression of ACP may well end up decreasing the fatty acid yields due to cellular toxicity. To circumvent some of the toxicity issues associated with the overexpression of ACP, we engineered artificially linked tandem ACP proteins as a means to enhance fatty acid yields in *E*. *coli*. We used a naturally occurring peptide linker from the tandem ACP multidomain PUFA synthase from *P*. *profundum* to link the *E*. *coli* ACP into di-domain (ACP_2_) and tri-domain (ACP_3_) fragments (Fig. 1b). Our results show the feasibility of enhancing fatty acid production by genetically fusing ACP domains together into multi-domain proteins.

## Results

### Protein purification and yield

First, the ACP_2_ and ACP_3_ fragments were made by overlap PCR, using primers that were complementary to both the *E*. *coli* gene encoding ACP and to the *P*. *profundum* gene encoding the peptide that links the ACPs from the PUFA synthase (Fig. S1). The resulting DNA fragments, *ACP2* and *ACP3*, were cloned into a pET100TOPO vector and expressed in *E*. *coli* BL21(DE3) cells. We purified the proteins by a combination of nickel-affinity purification and size exclusion chromatography, resulting in highly pure elution fractions with yields of 15, 24, and 18 mg of pure protein per liter of culture, for ACP_1_, ACP_2_, and ACP_3_, respectively.

### Size exclusion chromatography of fused ACPs

The relative size of the mono- di- and tri-domain proteins was determined using size exclusion chromatography. The retention time of the ACP protein was compared with the retention times of protein standards of known molecular weight. Chromatograms showed retention times corresponding to molecular weights of 21.1 kDa for ACP_1_, 36.7 kDa for ACP_2_, and 141.4 kDa for ACP_3_ when using a high salt buffer (Data not shown). The same analysis was conducted using a lower salt buffer to better reflect the low-salt conditions required for analyzing sample both by SAXS and by circular dichroism (CD). The size exclusion chromatogram resulted in retention times consistent with molecular weights of 30.0 kDa for ACP_1_, 95.3 kDa for ACP_2_, and 261.5 kDa for ACP_3_ (Fig. 1d). Under all conditions tested, the estimated molecular weights were found to be consistently higher than what would be expected based on the amino acid sequence of these proteins (Table 1). This observed increase in the apparent molecular weight was not the result of intermolecular disulfide bonds since neither ACP nor the amino acid linker contain cysteine residues within their amino acid sequence.

**Table 1:** Molecular weights of ACP proteins estimated by size exclusion chromatography at high salt (500 mM NaCl) and low salt (50 mM NaCl).

	Expected M.W. (kDa)	M.W. determined by SEC (kDa) (20 mM Tris, 500 mM NaCl)	M.W. determined by SEC (kDa) (20 mM NaH_2_PO_4_, 50 mM NaCl)
ACP_1_	12	21.1	30.0
ACP_2_	24	36.7	95.63
ACP_3_	37	141.4	261.5

### Solution structures of fused ACPs by SAXS

**Figure 2:**
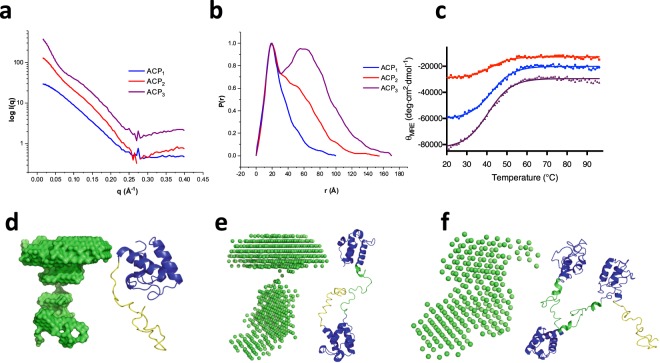
Solution structures of tandem ACPs by SAXS. The structure of ACP as a multidomain flexible tandem arrangement was confirmed by small-angle X-ray scattering (**a**) SAXS/WAXS curves were generated from the X-ray scattering data, and (**b**) a corresponding P(r) function was generated for each construct, from which a radius of gyration was obtained. (**c**) Further confirmation of the flexible arrangement in multi ACPs came from the thermal unfolding monitored at 222 nm between 20–95 °C. There was no significant difference in thermal unfolding between the constructs (40.9 °C ± 1.8 °C) indicating that ACP domains are not interacting. The SAXS data was used to generate three-dimensional structural models using DAMMIF within the ATSAS software package. The structures of (**d**) ACP_1_, (**e**) ACP_2_, and (**f**) ACP_3_ together with their corresponding homology model made using iTasser server. ACP domains are highlighted in blue, the amino acid linkers in green, and the His-tag in yellow.

To show that the engineered ACP constructs adopted a multi-domain arrangement, we gathered structural information by small-angle X-Ray scattering (SAXS). Figure 2a shows the scattering profiles for the three ACPs constructs. Indirect Fourier transformation of the scattering data yields a pair distribution function P(r), which is a real space representation of the scattering data and provides an approximation of the dimension and shape of a protein in solution. In this case, the ACP_1_ protein has a P(r) function that is relatively symmetric and bell-shaped, whereas the more flexible ACP_2_ and ACP_3_ have an extended tail with multiple shoulders and oscillations^17,18^. The P(r) functions also revealed gyration radii of 23.5 Å for ACP_1_, 36.6 Å for ACP_2_, and 48.0 Å for ACP_3_, all consistent with a flexible multi-domain arrangement (Fig. 2b). The maximum diameter of the proteins (D_max_) also increased as ACP domains were added, with D_max_ values of 100 Å for ACP_1_, 155 Å for ACP_2_, and 170 Å for ACP_3_. In terms of the protein shapes, the ACP_1_ P(r) function suggest a somewhat elongated structure, while those of ACP_2_ and ACP_3_ show new P(r) contours with two distinct peaks, a hallmark of flexible or dumbbell-like particles, consistent beads-on-a-string arrangements of domains (Fig. 2d–f).

### Thermal stability assays of fused ACPs

As a confirmation of the multi-domain nature of the artificially generated fragments, we measured the thermal denaturation using circular dichroism (CD) spectroscopy for all ACP constructs in this study. Results show that there is little difference in thermal stability between the three ACP constructs, suggesting that ACP linkage does not significantly impact stability, an observation consistent with domains that are structurally independent with little or no contact between ACP domains. The melting temperature (Tm) values were 42.3, 38.9, and 41.6 °C for ACP_1_, ACP_2,_ and ACP_3,_ respectively in an unfolding that was irreversible (Fig. 2c).

### Effect of fused ACPs on fatty acid production

**Figure 3:**
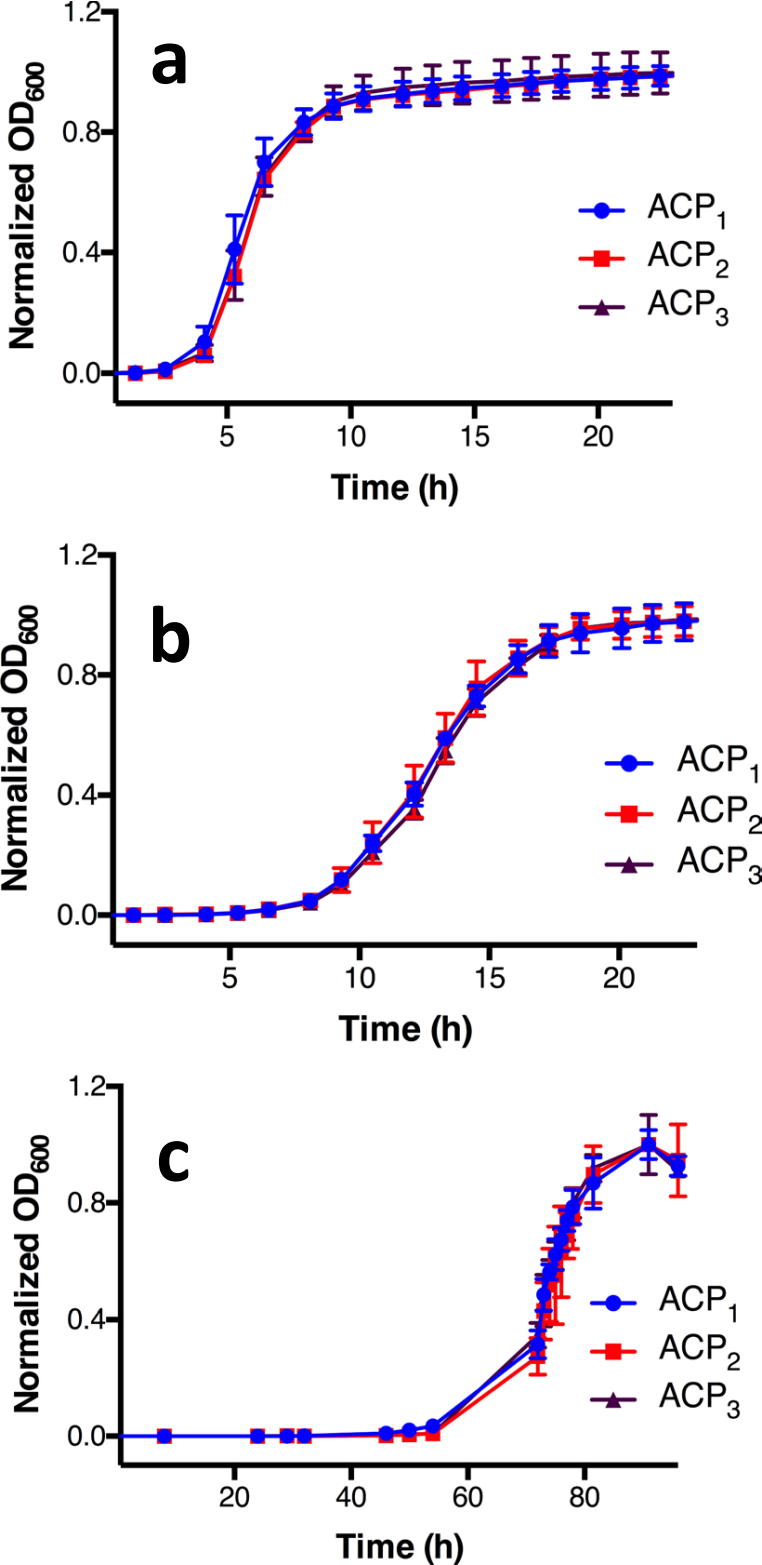
Effect of ACP expression on *E*. *coli* growth. Liquid cultures of *E*. *coli*. expressing either ACP_1_, ACP_2_ or ACP_3_ were grown at three different temperatures: (**a**) 37 °C, (**b**) 22 °C and (**c**) 15 °C. As expected, higher temperatures correlated with faster rates but the expression of ACP had no effect on growth rate. All experiments were carried out in triplicates and the error bars represent the standard deviation.

**Figure 4:**
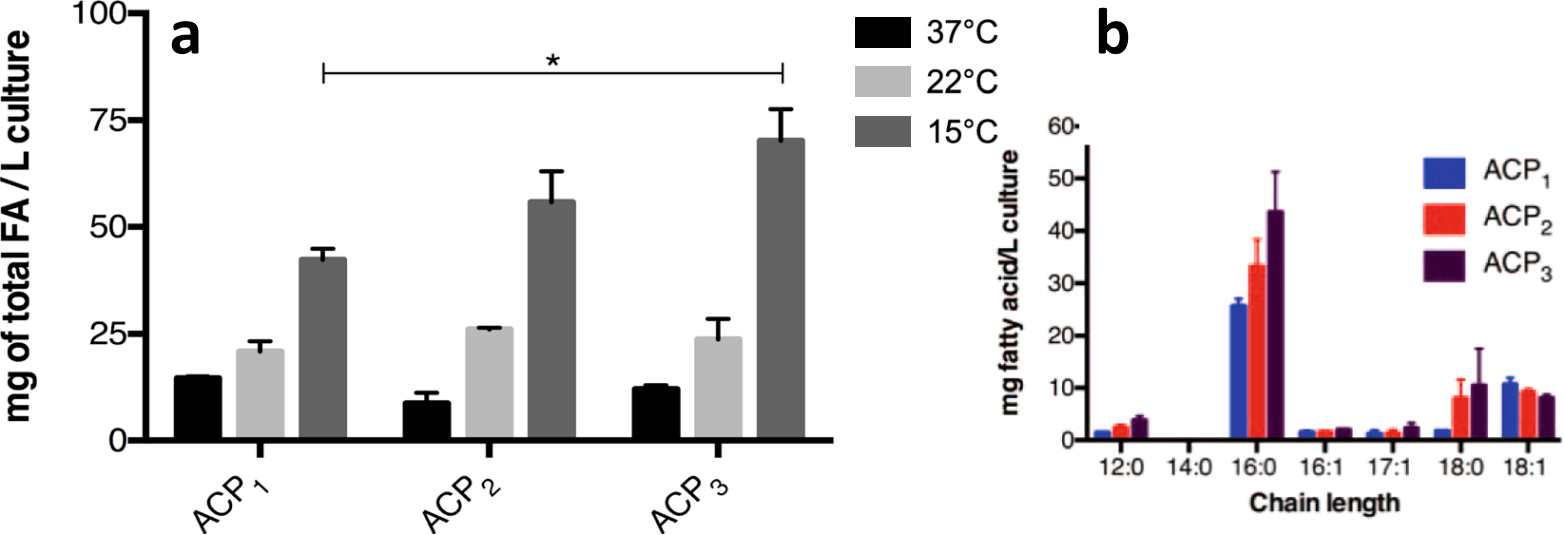
Total fatty acid yields of *E*. *coli* cultures expressing tandem ACPs. Fatty acids were extracted from the cultures and analyzed by GC/MS. (**a**) Cultures grown at 37 °C and 22 °C showed no significant effect of ACP expression on the production of fatty acids. At 15 °C, however, there was a significant effect of ACP on fatty acid production. (**b**) This increase FA production at 15 °C is primarily due to the production of saturated fatty acids 16:0 and 18:0. Cultures harboring ACP produced 42.3 ± 4.5 mg/L; for ACP2 the yield increased to 55.8 ± 12.6 mg/L, and the highest yield was observed for ACP_3_ at 70.2 ± 12.9 °C. Data are shown as means ± SEM and p -values were calculated using the Student’s t-test, p < 0.05. All samples were run as biological triplicates.

Cultures of *E*. *coli* expressing the different ACP constructs were grown at three different temperatures and their fatty acid content was analyzed by gas chromatography coupled to mass spectrometry (GC/MS). The growth rate of *E*. *coli* was unaffected by ACP expression at all temperatures, indicating that any effect on fatty acid production is not due to general metabolic impairment (Fig. 3). Higher fatty acid yields were obtained at the lowest temperature of 15 °C, followed by the cultures grown in 22 °C and 37 °C, respectively (Fig. 4). Cultures expressing the ACP_3_ at 15 °C showed higher fatty acid yield than cultures expressing ACP_2_ or ACP_1_, where the total FA yield was 42.3 mg/L (±3.1) for ACP_1,_ 55.8 mg/L (±8.9) for ACP_2_ and 70.2 mg/L (±9.1) for ACP_3_. This corresponds to a significant 1.6-fold enhancement for ACP_3_ over control ACP_1_. This enhancement was only observed at the lowest temperature, but not at 22 °C or 37 °C, a finding not totally unexpected since fatty acid production in *E*. *coli* is known to be suppressed at higher temperatures^16^.

## Discussion and Conclusion

The central role of ACP in the biosynthesis of fatty acids has invited explorations into their possible use to enhance fatty acid production in cultured bacteria. However, attempts to overexpress recombinant native ACP in *E*. *coli* have resulted in impaired cell growth, primarily by the inhibition of glycerol-3-phosphate acyl transferase^16^. A natural mechanism to control or enhance ACP availability is employed by PUFA-producing bacteria that enhance their levels of fatty acid production not by overexpressing ACP, but rather by harboring multiple copies of ACP along the same polypeptide in tandem arrangements^14,15^. In this work, we explored the technical feasibility of forcing the native *E*. *coli* ACP to form di-domain and tri-domain polypeptides: ACP-ACP (ACP_2_) and ACP-ACP-ACP (ACP_3_). The ACPs were fused together using a natural linker peptide sequence corresponding the spacer region between tandem ACP domains in the PfaA protein from *Photobacterium profundum*, a PUFA-producing organism. The growth curves of *E*. *coli* expressing ACP1, ACP2 and ACP3, reveal no toxicity for any of the ACP constructs, perhaps because the cultures were not induced with IPTG and thus, the ACPs were not overexpressed for this experiment.

Previous work by other groups had shown that the presence of multiple ACP domains in a single polypeptide enhances fatty acid production. In *Shewanella japonica* and other organisms, the production of eicosapentaenoic acid is proportional to the number of active ACP domains in the tandem arrangement. Conversely, the elimination of domains from the tandem arrangement results in a decrease in fatty acid production^14,15^. In this work, with the expression of the fused tandem ACPs, we observed a modest but significant gain in the production of fatty acids: 42 mg/L in cells expressing ACP_1_ to 70 mg/L in cells expressing ACP_3_, with no change in growth rate at 15 °C. This enhancement in fatty acid production was statistically insignificant at 22 °C and 37 °C. This temperature dependence was not totally unexpected since bacteria tend to decrease fatty acid production at higher temperatures. Thus it was expected that any effect on fatty acid production by ACP would be more pronounced at the lower temperature^19^.

Solution structures of the multi-ACP constructs show that they form an extended and highly flexible structure with little or no contact between domains^17,20^. A previous solution structure of the naturally occurring tandem ACPs from *P*. *profundum* revealed an elongated arrangement of quasi-independent domains, like beads on a string^9^. Similarly, the artificially fused tandem ACPs in this study also show a similar arrangement of independent domains. The thermal denaturation measured by circular dichroism shows that ACP_1_, ACP_2_ and ACP_3_ all have similar melting temperatures (Tm ACP_1_ = 42.3 °C vs. Tm ACP_2_ = 38.9 °C vs. Tm ACP_3_ = 41.6 °C). The fact that the presence of additional ACP domains does not further stabilize structures is consistent with a beads-on-a-string arrangement with little or no contact with neighboring or proximate ACP domains. Similarly, the size exclusion chromatography profiles for ACP_1_, ACP_2_ and ACP_3_ all suggest a structure that is larger than would be expected by the sequence (141 kDa vs 37 kDa by sequence for ACP_3_), which is consistent with an extended or a highly flexible arrangement of ACP domains^9^. The formation of higher-order oligomers by ACP_1_, ACP_2_ and ACP_3_ can be ruled out based on the SAXS-derived structures which show essentially monomeric extended and flexible structures (Fig. 2d–f).

In conclusion, we have shown that the incorporation of ACP domains into multi-ACP constructs can have a positive effect on fatty acid yield, while circumventing possible toxicity issues associated with the overexpression of ACP in *E*. *coli*. The multi-domain nature of the engineered constructs was confirmed using multiple biophysical methods including SAXS, circular dichroism and size exclusion chromatography. With the current interest in the production of microbially derived oils and materials, this strategy could be widely applicable to many microbial hosts used for the production of fatty acids and biodiesel.

## Methods

### Primer design of acpP

DNA sequence for *E*. *coli* DH10B *acpP* (Ref. sequence NC_000913.3:11516151151851) was obtained from NCBI. The linker region corresponded to amino acids 1511–1538 of the *pfaA* gene from *P*. *profundum* (Accession No. CAG19871.1). The primers for the amplification of *acpP* contained overhangs of additional sequence that are complementary to either the 5′ or 3′ terminus of the linker sequence (Table S1). The reverse primers for the amplification of *acpP* contained overhangs complementary the 5′ terminus of the linker sequence, whereas forward primers contained overhangs complementary to the 3′ terminus of the linker sequence. The same principle applied for the amplification of the *pfaA* linker. All primers were purchased from the RCMI Core Lab at UPR Medical Sciences Campus after verification of secondary structure and primer dimer formation using the online Thermo Scientific Multiple Primer Analyzer tool (www.thermoscientificbio.com/webtools/multipleprimer).

### Construction of gene fusions and cloning

A general scheme for the generation of fused tandem *acpP*s by overlap PCR is presented in Supplementary Fig. 1. All PCRs were done using PfuUltra II Fusion HS DNA Polymerase (Agilent). All PCR reactions were preceded by a denaturation step at 95 °C for two minutes and finished with a final extension step at 68 °C for 3 minutes. For the amplification of the DNA corresponding to the linker region of *pfaA*, 100 ng of plasmid DNA containing the ACP domains from *P*. *profundum* were mixed with primers 9 and 10. DNA was denatured at 95 °C for 1 minute, followed by annealing at 45.8 °C for 1 minute and finished with an extension 68 °C for 1 minute. For the amplification of *acpP*, approximately 2 ng of genomic DNA from *E*. *coli* DH10B cells were mixed with primers 1 and 4, using the same PCR parameters as for the tandem constructs. Three basic building blocks were constructed to either extend or terminate each tandem gene: an initiation unit (Table S2), an elongation unit (Table S4), and a termination unit (Table S3). The sequential PCR reactions were performed as outlined in Table S5. PCRs were run for 30 cycles without primers, and then 15 more cycles with primers. Individual PCR reaction products were separated by electrophoresis on an agarose gel (2%) and purified using the QIAQuick Gel Extraction Kit (Qiagen).

### Protein expression and purification

*E*. *coli* BL21(DE3) - Codon Plus RIL cells (Invitrogen) were transformed with each plasmid and grown in liquid Luria-Bertani medium supplemented with 0.4% glycerol, 1% glucose, which contained kanamycin (100 mg/L) and chloramphenicol (25 mg/ L). Cultures were grown at 37 °C, 250 rpm until OD_600_ = 0.2–0.3, at which point the temperature was lowered to 22 °C. Protein expression was induced with 1 mM IPTG once the OD_600_ reached 0.5–0.6. After 4 hours, the cells were harvested by centrifugation at 4 °C and 11000 × g on a Sorvall Lynx 4000 Centrifuge using a Fiberlite™ F14-14 × 50cy Fixed-Angle Rotor (Thermo). Samples were stored at −20 °C overnight. Pellets were resuspended in lysis buffer (20 mM Tris, 500 mM NaCl, 1 mM DTT, 20% glycerol, pH 7.8) in the presence of lysozyme (10 mg/ml), DNAse (1 mg/ml), 2X protease inhibitor cocktail (Pierce) and sonicated. The lysates were collected by centrifugation (11000 × g, 4 °C, 30 min). The soluble lysates were poured through a column filled with Ni-Sepharose (Sigma) that had been equilibrated with the corresponding buffer +5 mM imidazole, and washed twice with the same buffer +10 mM imidazole. His-tagged proteins were eluted in the in the corresponding buffer containing 200 mM imidazole. Afterwards, fractions were infused into a size exclusion chromatography Superdex 200 Increase 10/300 GL column (GE Healthcare) equilibrated in the NiNTA lysis buffer minus glycerol, and eluted at a flowrate of 0.8 mL/min. Purities of elution fractions were analyzed by SDS-PAGE. Protein yields were calculated using Nanodrop A280 quantification and dividing total milligrams of protein per volume of culture.

### Bacterial growth curves

After inocculation for protein production, 150 μL of the inoculated media were transferred to a Falcon 96 flat bottom plate and growth curves at 37 and 22 °C were generated using a Synergy H1 plate reader by monitoring absorbance at 600 nm as a function of time. Equipment configuration was as follows: normal read speed with a 100 msec delay, continuous orbital shake at a frequency of 237 cpm. The total runtime was 27 hours with a 10 minute interval between reads. Due to equipment limitations, the samples analyzed at 15 °C were grown inside a shaker incubator at the same agitation speed and the absorbance was then measured inside the plate reader. Because of the delayed growth, the total runtime was 96 hours.

### Determination of molecular weights by size exclusion chromatography

The purified proteins were exchanged into a low-salt system (20 mM NaH_2_PO_4_, 50 mM NaCl, pH 7.8) and run as in the purification step. Each run with the proteins was preceded by a run with a mixture of standard proteins (GE Healthcare) [aprotinin (6,500 Da), ribonuclease (13,700 Da), ovalbumin (44,000 Da), conalbumin (75,000 Da), aldolase (158,000 Da), ferritin (440,000 Da)] to generate a K_av_ vs. logMW curve, where K_av_ = (V_e_ − V_o_)/(V_c_ − V_o_). Proteins were eluted at a flowrate of 0.8 mL/min, and the elution volumes were determined using the Unicorn software integration function. The resulting standard curve was used to estimate the molecular weight for our proteins.

### Thermal stability determination by circular dichroism

Proteins were exchanged into 20 mM NaH_2_PO_4_, 50 mM NaCl, pH 7.8 using size exclusion chromatography. All samples came from new aliquots that had not been previously thawed and refrozen. Samples were diluted to 0.1 mg/mL and placed in a Jasco 1 mm quartz cuvette. Thermal denaturation was measured by monitoring the CD signal at 218 nm as a function of temperature in the range of 20–95 °C (1 °C/min) in a Jasco-1500 spectropolarimeter using the Interval Temperature Scan Measurement program. Precipitation was observed at the end of the run in every sample. Parameters for Tm scan were: CD scale 200mdeg/1.0dOD, D.I.T 1 s, bandwidth 1 nm. CD units (mdeg) were converted to θ_MRE_ units using the equation θ_MRE_ = (deg*MRW)/(10*c*b), where MRW is the mean residue weight (Da), c is the enzyme concentration (g/mL), and b is the cell pathlength (cm). Denaturation analysis was performed using the built-in Thermal Denaturation Analysis Program.

### Solution and in silico structures using small angle X-Ray scattering and I-tasser

Proteins were exchanged into 20 mM NaH_2_PO_4_, 50 mM NaCl, pH 7.8 and concentrated between 1–3 mg/mL. X-ray scattering measurements were conducted at the life sciences X-ray scattering (LiX) beamline at the National Synchrotron Light Source II at Brookhaven National Laboratory, Brookhaven, NY. About 10 μl of the enzymes and buffer solutions were continuously flowed through a capillary and exposed to the X-ray beam for 5 s. The measurements were carried out in triplicate. Data processing was performed using an automated Python-based package developed at LiX. Overall, the two-dimensional scattering patterns from protein solutions were first recorded on 3 detectors simultaneously and then converted into one-dimensional scattering profiles. The SAXS/WAXS data were then merged, averaged, and buffer subtracted to obtain relative scattering intensity (I) as a function of the momentum transfer vector, q (q = (4πsinθ)/λ), where λ is the beam wavelength, and θ is the scattering angle. The resulting combined and merged scattering pattern was analyzed using PRIMUS, GNOM, and DAMMIF of the ATSAS software package^21–23^. The Rg value derived from the Guinier analysis corresponded well to the Rg obtained through the indirect transform algorithm in GNOM. Theoretical three-dimensional models of the ACPs were generated using the I-TASSER algorithm, which builds protein models from primary sequences by combining remote homologs with *ab initio* calculations^24^. Pymol was used for graphical analysis and figure generation.

### Extraction and modification of fatty acids for quantification

Cultures were grown in triplicate in LB media using the same procedure as before at 37, 22, and 15 °C, in the absence and presence of 1 mM IPTG. After a total of 27 h, cells were harvested, frozen at −80 °C, and lyophilized. Fatty acids were extracted using the Bligh-Dyer method^25^. Briefly, dried pellets were dissolved in NaCl, chloroform, MeOH, and vortexed. The organic phase was isolated after centrifugation and concentrated under N_2_. Finally, the concentrated fatty acids were resuspended in a mixture of chloroform:MeOH (2:1). To convert fatty acids to their respective methyl esters, the extracted fatty acids in the chloroform:MeOH mixture were reacted with methanolic HCl and refluxed for 2 h under constant stirring and heating. Methyl heneiocosanoate was added as an internal standard prior to the addition of methanolic HCl. Fatty acids were isolated by extraction with hexane, dried under N_2_, and resuspended in 200 μL of hexane.

### Quantification of fatty acids and statistical analysis

Fatty acid profiling was performed using a GC/MS-QP2010 (Shimadzu) equipped with a fused-silica FAMEWAX capillary column (30 m × 0.32 mm i.d. × 0.25 μm film thickness) (Restek) and an auto-injector (Shimadzu, AOC-20i). Samples were injected in the split mode (spit ratio = 15:1). The oven temperature was set to 130 °C for 5 min, followed by a temperature ramp from 130 to 250 °C at 4 °C/min, and finally, held at 250 °C for 5 min, using helium as the carrier gas. Mass spectra data were obtained after electron impact ionization (EI, 70 eV, ion source temperature 200 °C) in full scan mode between 50 and 600 amu. The temperatures of the injection port and the detector were set at 250 °C. The data was processed using the GC/MS LabSolutions Postrun Analysis software (Shimadzu) for metabolite identification using the NIST 2014 spectral mass library (National Institute of Standards and Technology). Fatty acid yields were calculated as follows: First, the millimoles of each fatty acid were calculated using the formula mmol FA = (A_FA_/A_IS_)*C_IS_*V_chl_, where (A_FA_/A_IS_) is the ratio of the areas of each fatty acid and the internal standard calculated from the gas chromatogram, C_IS_ is the known concentration of the internal standard (M), and V_chl_ is the volume of chloroform:MeOH used to resuspend the concentrated fatty acids (mL). From here, the milligrams of each fatty acid per liter of culture were calculated as mg FA/L = (mmol FA*g_cell_*MW)/(g_ext_*V_tot_), where g_cell_ is the total dry cell mass (g), g_ext_ is the dried cell mass used for the extraction (g), V_tot_ is the total culture volume (L), and MW is the molecular weight for each fatty acid (g/mol). Individual fatty acid yields were pooled in order to determine the amount of total fatty acids per liter of culture. Statistical analysis was performed using GraphPad Prism 6. Data are shown as means ± SEM and p -values were calculated using the Student’s t-test, p < 0.05.

## Supplementary information

Supplementary Material
