# Supplementary material for: Artificial covalent linkage of bacterial acyl carrier proteins for fatty acid production
Source: Sci Rep. 2019 Nov 5;9:16011. doi: 10.1038/s41598-019-52344-w (PMC6831569; doi:10.1038/s41598-019-52344-w)
Supplement: Supplementary file 1 — Supplementary Material [file 41598_2019_52344_MOESM1_ESM.pdf]

Supplementary Material for:

Artificial covalent linkage of bacterial acyl carrier proteins for fatty acid production

Carlos Rullán-Lind[1,2], Melissa Ortiz-Rosario[1,2], Andrea García-González[1,2],Vivian Stojanoff[3], Nataliya E. Chorna[1], Ruth B. Pietri[4], Abel Baerga-Ortiz[1,2]

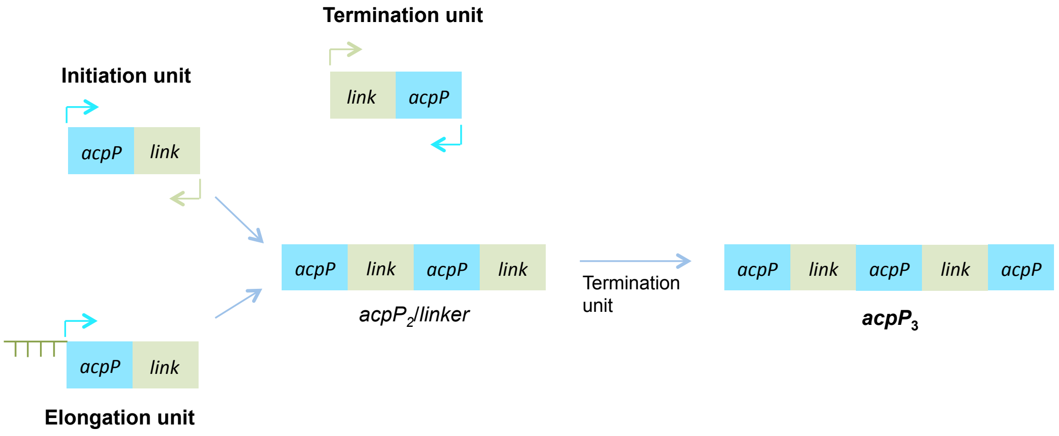

Supplementary Figure 1. Strategy for the construction of fused tandem ACPs. The primers for the amplification of *acpP* contained additional sequence complementary to either the 5’ or 3’ terminus of the sequence for the *pfaA* linker. Genes amplified using the overhanging forward primer contained additional sequence complementary to the 3’ end of the linker gene. Those genes amplified using the overhanging reverse primer contained additional sequence complementary to the 5’ end of the linker gene. Combining both of these products in a PCR reaction will anneal both genes forming an *acpP-linker* (or *linker-acpP*) intermediate. Three intermediates were produced: an initiation unit, an elongation unit, and a termination unit. The initiation unit contains a 5’CACC overhang sequence to facilitate the cloning into pET200/D-TOPO. From this point, the intermediates could either be extended by a subsequent PCR reaction with an elongation unit or prepared for cloning using a termination unit. The terminator unit contains a 3’TTA encoding a stop codon.

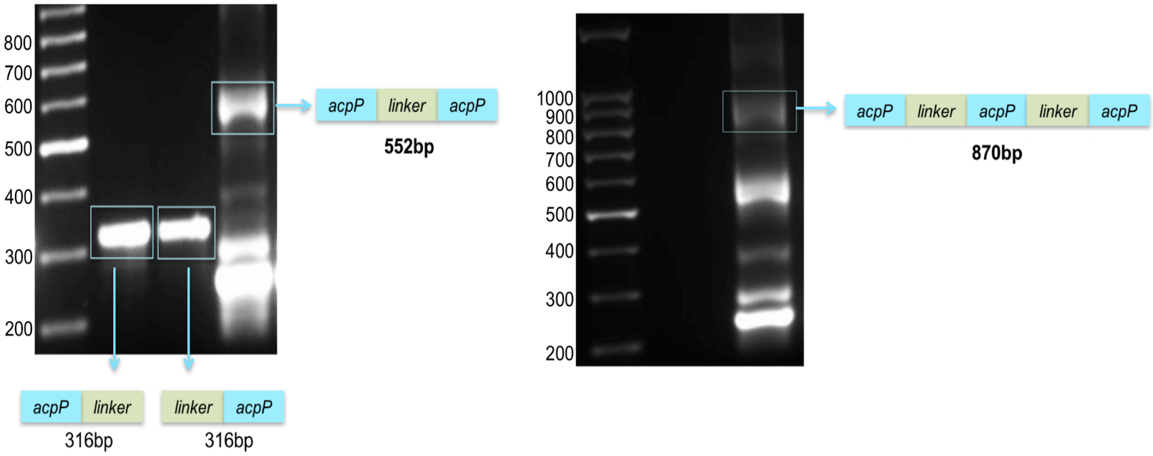

Supplementary Figure 2. Generation of tandem acpP gene constructs. Gene fusions were visualized on a 2% agarose gel. A band in the 552bp region was observed corresponding to *acpP*$_2$. The 552 base pairs can be accounted for by the base pairs corresponding to the linker (84bp) plus the base pairs corresponding to two *acpP* sequences (234bp each). *acpP*$_3$ was observed as a band in the 870bp region.

Table S1. Primers for the amplification of acpP fragments and the linker fragment from pfaA. A list of all the oligonucleotide primers used for the construction of the tandem ACP fragments in this study. In the following tables, oligonucleotides are named using these numbers. Sequences in bold are overhangs corresponding to either the CACC insertion sequence for TOPO cloning or the 3' and 5' sequences of the linker peptide sequence.

Primer #	Primer Name	Sequence (5’ → 3’)
**1**	*acpP* Fwd TOPO	**CACC**ATGAGCACTATCGAAGAACGC
**2**	*acpP* Rv + Overhang	**TACATTTTGTGTAGA**CGCCTGGTGGCCGTTG
**3**	*acpP* Fwd + Overhang	**AAGTTCAAGGCACA**ATGAGCACTATCGAAGAACGC
**4**	*acpP* Rv + Stop	**TTA**CGCCTGGTGGCCGTTG
**5**	*pfaA* Fwd	TCTACACAAAATGTAGCGATTC
**6**	*pfaA* Rv	TGTGCCTTGAACTTGTGCTG

Table S2. PCR conditions used for the production of the initiation unit. Two PCR reactions were used to generate the initiation unit consisting of (i) the ACP gene fused to (ii) the gene encoding the linker peptide.

<table>
<tr><th>Product</th><th colspan="3">Reaction I</th><th colspan="3">Reaction II</th></tr>
<tr><td rowspan="9">Initiation unit</td><td colspan="3">CACC-acpP-Overhang</td><td colspan="3">Initiaton unit</td></tr>
<tr><td>Template(s)</td><td>Fwd.</td><td>Rv.</td><td>Template (s)</td><td>Fwd.</td><td>Rv.</td></tr>
<tr><td>DH10B genomic</td><td>1</td><td>2</td><td>Rxn I + linker</td><td>1</td><td>6</td></tr>
<tr><td>Expected bp</td><td colspan="2">250</td><td>Expected bp</td><td colspan="2">318</td></tr>
<tr><td colspan="3">PCR Parameters</td><td colspan="3">PCR Parameters</td></tr>
<tr><td>Step</td><td>ºC</td><td>Time</td><td>Step</td><td>ºC</td><td>Time</td></tr>
<tr><td>Denaturation</td><td>95.0</td><td>2:00</td><td>Denaturation</td><td>95.0</td><td>2:00</td></tr>
<tr><td>Annealing</td><td>48.4</td><td>1:00</td><td>Annealing</td><td>46.3</td><td>1:00</td></tr>
<tr><td>Extension</td><td>68.0</td><td>1:00</td><td>Extension</td><td>68.0</td><td>1:00</td></tr>
</table>

Table S3. **PCR conditions used for the production of the termination unit**. Two PCR reaction were used to generate the termination unit consisting of (i) the gene encoding the linker peptide fused to (ii) the ACP gene.

<table>
<tr><th>Product</th><th colspan="3">Reaction I</th><th colspan="3">Reaction II</th></tr>
<tr><td rowspan="9">Termination unit</td><td colspan="3">Overhang-acpP-TTA</td><td colspan="3">Termination unit</td></tr>
<tr><td>Template(s)</td><td>Fwd.</td><td>Rv.</td><td>Template (s)</td><td>Fwd.</td><td>Rv.</td></tr>
<tr><td>DH10B genomic</td><td>3</td><td>4</td><td>Rxn I + linker</td><td>5</td><td>4</td></tr>
<tr><td>Expected bp</td><td colspan="2">248</td><td>Expected bp</td><td colspan="2">318</td></tr>
<tr><td colspan="3">PCR Parameters</td><td colspan="3">PCR Parameters</td></tr>
<tr><td>Step</td><td>ºC</td><td>Time</td><td>Step</td><td>ºC</td><td>Time</td></tr>
<tr><td>Denaturation</td><td>95.0</td><td>2:00</td><td>Denaturation</td><td>95.0</td><td>2:00</td></tr>
<tr><td>Annealing</td><td>48.4</td><td>1:00</td><td>Annealing</td><td>46.3</td><td>1:00</td></tr>
<tr><td>Extension</td><td>68.0</td><td>1:00</td><td>Extension</td><td>68.0</td><td>1:00</td></tr>
</table>

Table S4. **PCR conditions used for the production of the elongation unit**. Two PCR reaction were used to generate the termination unit consisting of (i) the ACP gene flanked on both ends by (ii) genes encoding the linker peptide

Product	Reaction I			Reaction II		
**Elongation unit**	***Overhang-acpP-Overhang***			**Elongation unit**		
	**Template(s)**	**Fwd.**	**Rv.**	**Template (s)**	**Fwd.**	**Rv.**
	DH10B genomic	3	2	Rxn I + linker	3	6
	Expected bp	264		Expected bp	332	
	**PCR Parameters**			**PCR Parameters**		
	**Step**	**ºC**	**Time**	**Step**	**ºC**	**Time**
	Denaturation	95.0	2:00	Denaturation	95.0	2:00
	Annealing	45.8	1:00	Annealing	46.3	1:00
	Extension	68.0	1:00	Extension	68.0	1:00

Table S5. PCR conditions used for the production of ACP2 and ACP3. The products of different previous reactions (initiation, elongation and termination units) were mixed with the corresponding oligonucleotides to yield the final PCR products.

acpP$_2$			***acpP***$_3$					
**Reaction I**			**Reaction I**			**Reaction II**		
**Template(s)**	**Fwd.**	**Rv.**	**Template (s)**	**Fwd.**	**Rv.**	**Template (s)**	**Fwd.**	**Rv.**
Init. + Term.	1	4	Init. + Elon.	1	6	Rxn I + Term.	1	4
Expected bp	552		Expected bp	636		Expected bp	870	
**PCR Parameters**			**PCR Parameters**			**PCR Parameters**		
**Step**	**ºC**	**Time**	**Step**	**ºC**	**Time**	**Step**	**ºC**	**Time**
Denaturation	95.0	1:00	Denaturation	95.0	1:00	Denaturation	95.0	1:00
Annealing	58.1	1:00	Annealing	45.8	1:00	Annealing	45.8	1:00
Extension	68.0	1:00	Extension	68.0	1:00	Extension	68.0	1:00
